# Single and Synergistic Effects of Cannabidiol and Δ-9-Tetrahydrocannabinol on Zebrafish Models of Neuro-Hyperactivity

**DOI:** 10.3389/fphar.2019.00226

**Published:** 2019-03-20

**Authors:** Éric Samarut, Jessica Nixon, Uday P. Kundap, Pierre Drapeau, Lee D. Ellis

**Affiliations:** ^1^ Department of Neurosciences, Research Center of the University of Montreal Hospital Center (CRCHUM), Université de Montréal, Montréal, QC, Canada; ^2^ Modelis Inc., Montréal, QC, Canada; ^3^ National Research Council of Canada, Halifax, NS, Canada

**Keywords:** neuro-hyperactivity, cannabis, cannabinoids, zebrafish, seizures

## Abstract

In this study, we aimed to investigate the effect of the two main active cannabinoids extracted from cannabis: Δ-9-tetrahydrocannabinol (THC) and cannabidiol (CBD) on two distinct behavioral models of induced neuro-hyperactivity. We have taken advantage of two previously developed zebrafish models of neuro-hyperactivity: a chemically induced pentylenetetrazole model and a genetic model caused by loss-of-function mutations in the GABA receptor subunit alpha 1 (*GABRA1−/−*). Both CBD and THC have a significant effect on the behavioral changes induced by both models. Importantly, we have also shown that when applied together at different ratios of THC to CBD (1:1, 1:5, and 1:10), there was a synergistic effect at a ratio of 1:1. This was particularly important for the genetically induced neuro-hyperactivity as it brought the concentrations of THC and CBD required to oppose the induced behavioral changes to levels that had much less of an effect on baseline larval behavior. The results of this study help to validate the ability of THC and CBD to oppose neuro-hyperactivity linked to seizure modalities. Additionally, it appears that individually, each cannabinoid may be more effective against the chemically induced model than against the *GABRA1*−/− transgenic model. However, when applied together, the concentration of each compound required to oppose the *GABRA1*−/− light-induced activity was lowered. This is of particular interest since the use of cannabinoids as therapeutics can be dampened by their side-effect profile. Reducing the level of each cannabinoid required may help to prevent off target effects that lead to side effects. Additionally, this study provides a validation of the complimentary nature of the two zebrafish models and sets a platform for future work with cannabinoids, particularly in the context of neuro-hyperactivity disorders such as epilepsy.

## Introduction

The number of disease states linked to neuro-hyperactivity is large ranging from numerous psychiatric and muscular disorders to seizures and epilepsy. Numerous disease models of altered neuroactivity, such as anxiety, epilepsy, bi-polar disorder, and schizophrenia, have been developed using zebrafish (Ai et al., 2001; Baraban et al., 2005, 2007; Morris, 2009; Maximino et al., 2010; Seibt et al., 2010, 2011; Hortopan et al., 2010b; Brennan, 2011; Souza and Tropepe, 2011; Zakhary et al., 2011; Ellis et al., 2012; Ellis and Soanes, 2012). Importantly, it has been demonstrated that the aforementioned models can be used as high-throughput, cost-effective systems for screening various potential therapeutics. With respect to epilepsy, a number of seizure models have been developed, which include both chemically induced and transgenic models (Baraban et al., 2005, 2013; Baraban, 2007; Hortopan et al., 2010b; Ellis et al., 2012; Samarut et al., 2018). The most well characterized chemically induced model is the pentylenetetrazole (PTZ) exposure model. It has been demonstrated that zebrafish larvae exposed to PTZ show a concentration-dependent change in behavior that begins as an increase in swimming behavior, followed by fast darting activity and finally clonic convulsions accompanied by a loss of posture (Baraban et al., 2005). Importantly, in addition to the changes in behavior, exposure to PTZ leads to electrophysiological changes consisting of small amplitude discharges reminiscent of interictal bursts. This is accompanied by regionalized elevations in neuronal activity as measured through an increased expression of the immediate early gene *c-fos* (Baraban et al., 2005; Ellis et al., 2012). It then appears that the behavioral changes induced by PTZ exposure may be the consequence of an increase in neural activity that can be considered seizure-like in nature.

While chemically induced seizure models including PTZ have classically been used in various animal models to screen potential anti-epileptic compounds, one of their shortcomings stems from the fact that the seizures produced do not represent the spontaneous, recurring seizures that are required to be considered epileptic (Scheffer et al., 2017). One of the advantages of using zebrafish as a model system is their genetic tractability and the rapidly evolving technologies for generating transgenic models. It has been demonstrated that targeted knockout of either sodium channels or GABA receptors can result in spontaneous seizure events in larvae (Hortopan et al., 2010a,b; Baraban et al., 2013; Samarut et al., 2018). One of these models is the recently developed *GABRA1* knockout fish that produce sporadic seizures that are associated with intense and generalized neuronal activity thought to resemble tonic-clonic generalized idiopathic epilepsy in humans (Samarut et al., 2018). This genetic model offers a unique advantage for drug-screen purposes since in addition to the sporadic seizures, seizures can be triggered by exposure to light. Since zebrafish provide a high-throughput, cost-effective screening platform (Kundap et al., 2017), testing potential therapeutics against multiple seizure models concurrently (chemically induced and genetic epilepsies) is reasonable and has the potential to compliment the pre-clinical rodent models currently in use by providing additional information on the efficacy of novel anti-epileptic drugs (AEDs) (Maljevic et al., 2017).

While the treatment of seizures is in fact one of the oldest reported uses of cannabis (Friedman and Sirven, 2017), for the past century, the prohibition of cannabis has led to general opposition to its use as a therapeutic. Importantly, the use of cannabis and cannabinoids to treat numerous diseases has recently begun to gain general acceptance. However, with respect to epilepsy, there has been significant pushback against the use of cannabinoids with some studies, suggesting there is no concrete evidence proving their efficacy (Abrams, 2018). This opposition generally stems from a lack of adequate clinical trials and a lack of knowledge of the mechanism of action of the different cannabinoids contained within the plant (Perucca, 2017). Although some synergistic effects between CBD and THC for treating epilepsy have been speculated in a clinical trial (Tzadok et al., 2016), there is no *in vivo* evidence of the efficacy of the combined effect in comparison to single exposure. Lastly, cannabinoids as therapeutics are rarely used alone but are generally used as adjuncts to other standard prescription medications as is generally the case with epilepsy. The use of cannabinoids as adjunct therapeutics further complicates the assessment of their efficacy for the treatment of epilepsy in the clinical setting. In order to study the use of cannabinoids for the treatment of epilepsy, *in vivo* testing that can provide information on not only their efficacy but also their potential interaction with other anti-epileptic compounds is required.

Here we took advantage of the two aforementioned zebrafish seizure models, the chemically induced PTZ model and the *GABRA1* loss-of-function genetic model to test and compare the single and combined efficacy of the two major phytocannabinoids. We have shown that both THC and CBD appear to oppose some aspects of each model, and importantly, their combined use leads to a synergistic effect that is most evident for the genetically induced neuro-hyperactivity.

## Materials and Methods

### Fish Husbandry and Fish Lines

Zebrafish (*Danio rerio*) were maintained according to standard animal care protocols (Westerfield, 1995, 2000), in accordance with the Canadian Council of Animal Care (CCAC) guidelines and conducted at either the National Research Council of Canada or the Research Center of the University of Montreal Hospital (CRCHUM). Adult AB/Tubingen zebrafish, both wild-type and *GABRA1* knockout (Samarut et al., 2018), were housed on a re-circulating aquatic system at 28.5 ± 1°C, pH 7.0–7.2 on a 14:10 h light:dark schedule. Embryos from multiple breeding pairs were collected and pooled in E3 media (5 mM NaCl, 0.17 mM KCl, 0.33 mM CaCl_2_-2H_2_O, 0.33 mM MgSO_4_-7H_2_O, 10 mM HEPES, and pH 7.2) for 4–6 h. Following incubation, unfertilized embryos were removed, and the remaining embryos were housed in Pentair aquatic Eco-system nursery baskets (200 embryos per basket) in 3 L Tecniplast tanks, with matching conditions to adults.

### Larval PTZ Seizure Assay

At 5 days post-fertilization (dpf), zebrafish larvae were transferred to a 48-well plate with 1 larvae per well in 400 μl of buffered E3 media using a micropipette and acclimated for 1 h at 28°C in a light incubator. Pure cannabinoids were stored as a 1 mg/ml methanol stock solution at −20°C. Working solutions were prepared fresh each day at a 10× concentration from a fresh 100 μg/ml solution. About 50 μl of the 10× cannabinoid solution was pipetted into each well to reach the desired concentration and acclimated for 1 h in the light incubator. About 50 μl of a 10× PTZ solution was added to each well, and plates were directly loaded into the automated behavioral tracking system.

### Video Tracking and Analysis

For the PTZ model, behavior was testing using either a Noldus DanioVision or a Viewpoint Zebrabox system as a lightproof recording chamber with an infrared camera. Behavior was assessed using Zebralab (Viewpoint) and Ethovision XT13 (Noldus) software. The standard measure of activity was the total distance traveled quantified into either 60-s or 30-min bins. Each experimental replicate was comprised of 12 larvae per treatment group. The assay was completed a minimum of three times for each concentration of extract, and the replicates were compared (one-way ANOVA, 95% confidence interval). Pending no significant differences between groups, larval activity was pooled for each treatment group and all the controls as a single group to allow for further analysis. In addition to the total distance traveled, the distance traveled at a velocity above 20 mm/s was used as a measure of “fast” activity. This threshold was based on previously established protocols (Ellis et al., 2012) and is classified as “fast” activity as it is a threshold that is rarely exceeded by control larvae (<5% of total movement). The difference in activity between treatment groups and controls was assessed using a one-way ANOVA followed by Dunnet’s multiple comparison test, *p* < 0.05.

### Larval Genetic Seizure Model

A Noldus DanioVision set up was used as a lightproof recording chamber with an infrared camera. Seizures were induced after 1 h spent in darkness by switching on the build-in cold-white LED light for at least 1 min. Ethovision XT12 was used for analyzing the distance swam and maximum acceleration as well as to extract swimming tracks. A minimum of 15 larvae were tested from three independent replicates. Based on previous testing (Samarut et al., 2018), the effect of treatment was compared to the −/− gabra1 control levels using Student’s *t* test, *p* < 0.05. For the co-exposure experiments, the data were compared using a one-way ANOVA (*p* < 0.05).

## Results

### Pre-exposure to Either CBD or THC Opposes PTZ-Induced Neuro-Hyperactivity

**Figure 1:**
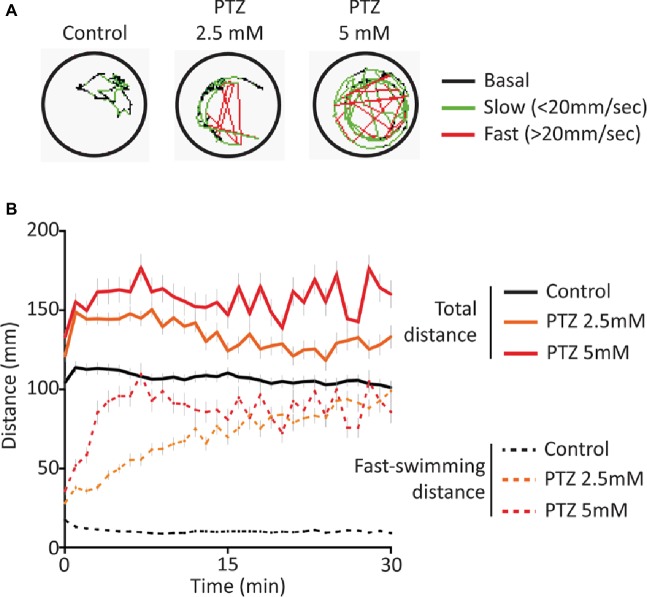
Behavioral response following acute exposure to PTZ. **(A)** Representative behavioral trace for 60 s from a single larvae. **(B)** Larval activity pattern as measured by average distance traveled (60 s time bins) immediately after introduction of PTZ (*n* = 144 for PTZ 2.5, *n* = 108 for PTZ 5, *n* = 252 for controls).

Exposure to both 2.5 and 5 mM PTZ produced an increase in baseline activity that was accompanied by an increase in sporadic fast-darting activity (Figure 1) that is considered an indicator of neuro-hyperactivity (Griffin et al., 2018). Following acute exposure to PTZ, the overall activity of the larvae rapidly reached a plateau and remained elevated for extended periods of time. The fast-darting activity also reached a plateau; however, larvae exposed to 2.5 mM PTZ required a longer period of time to reach this plateau level than those exposed to 5 mM PTZ indicating a concentration-dependent effect of PTZ exposure (Figure 1B).

**Figure 2:**
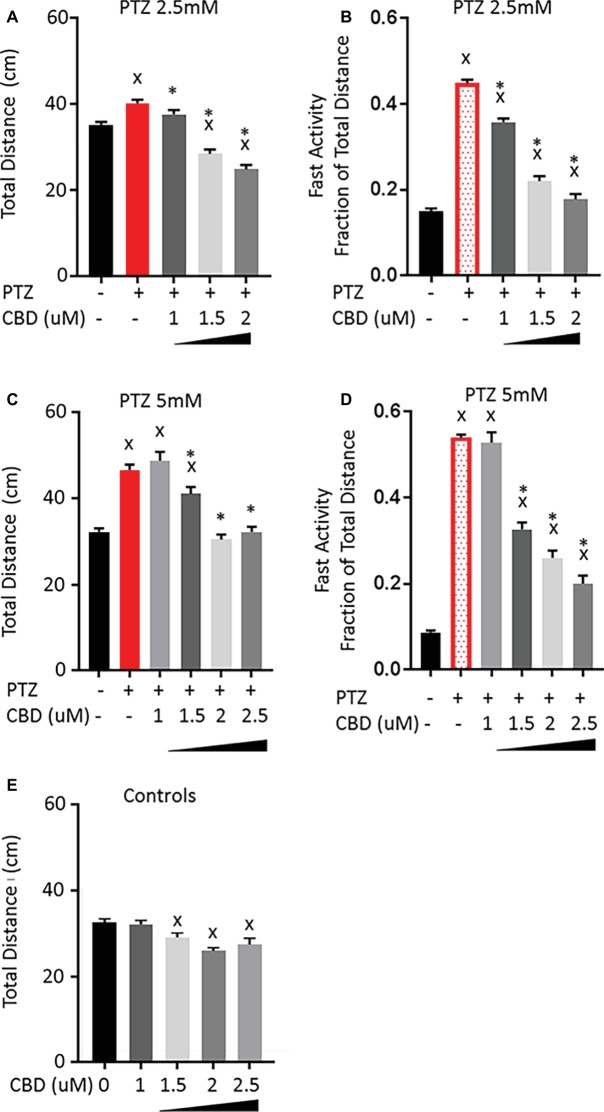
Comparison of PTZ-induced activity patterns following pre-exposure to cannabidiol (CBD). **(A,C)** Total activity during 30-min time bin following exposure to 2.5 and 5 mM PTZ. **(B,D)** Fast activity (>20 mm/s) represented as a fraction of total activity. **(E)** Control activity following CBD exposure. Significance was measured using a one-way ANOVA followed by Dunnet’s multiple comparison test (*p* = 0.05). X: significant difference from controls.*: significant difference from PTZ (*n* = 36 for each treatment group; *n* = 108 for PTZ 2.5 controls; and *n* = 144 for PTZ 5 controls).

Based on our previous work with CBD and THC (Achenbach et al., 2018), we chose a narrow concentration range beginning at the minimal observable effect level following acute exposure and increased the concentration until a significant effect on the PTZ-induced activity pattern was evident. Larvae were treated with CBD or THC for 1 h before PTZ was administered, and their activity was quantified for the first 30 min following PTZ exposure. Pretreatment of larvae with 1 μM CBD did not affect the increase in total activity but led to a small decrease in the fast activity produced by 2.5 mM PTZ (Figures 2A,B). Increasing the concentration of CBD led to a concentration-dependent decrease in the total activity and a further decrease in the fast activity produced by 2.5 mM PTZ. When the level of PTZ was increased to 5 mM, 1 μM CBD was no longer able to lower the fast activity, however, increasing the concentration of CBD led to a concentration-dependent reduction in both the total and the fast-activity induced by 5 mM PTZ (Figures 2C,D). It should be noted that while CBD was able to reduce the total activity to levels that were not significantly different than controls, the fast-activity level remained slightly elevated. CBD produced a significant reduction in the total activity of control larvae; however, this reduction was small, which is consistent with the previous work from our group that showed similar baseline activity for CBD-treated larvae to controls at concentrations higher than what was tested here (Figure 2E; Achenbach et al., 2018). This, along with the fact that the total activity of the PTZ-treated larvae was returned to levels similar to controls, indicates that the effect of CBD is not merely due to sedation, but rather it specifically opposes the fast-darting activity while having a minimal effect on the underlying normal movements.

**Figure 3:**
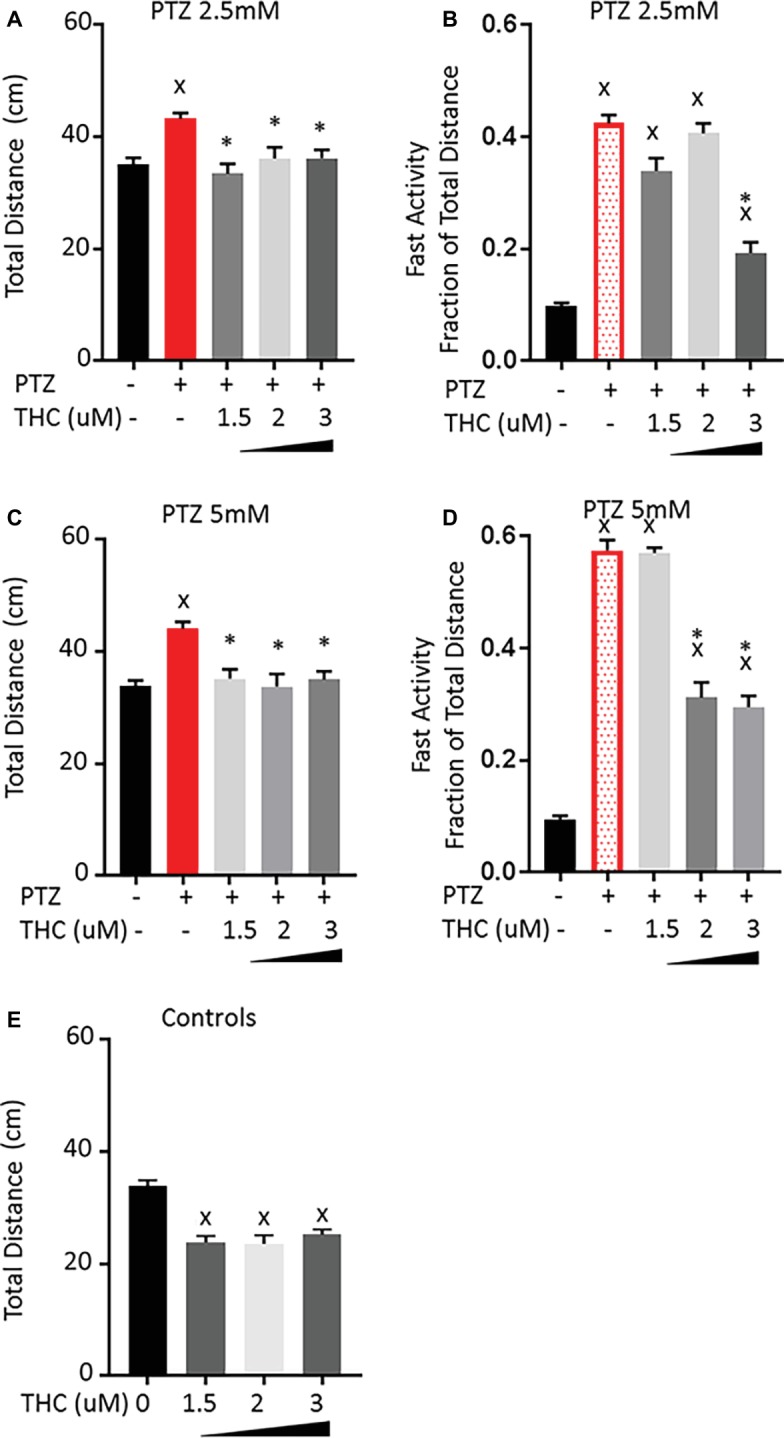
Comparison of PTZ-induced activity patterns following pre-exposure to tetrahydrocannabinol (THC). **(A,C)** Total activity during 30-min time bin following exposure to 2.5 and 5 mM PTZ. **(B,D)** Fast activity (>20 mm/s) represented as a fraction of total activity. **(E)** Control activity following THC exposure. Significance was measured using a one-way ANOVA followed by Dunnet’s multiple comparison test (*p* = 0.05). X: significant difference from controls.*: significant difference from PTZ (*n* = 36 for each treatment group; *n* = 108 for PTZ 2.5, PTZ 5, and controls).

The effect of pre-treatment with THC showed different effects on the PTZ-induced behavioral patterns than CBD. For all three concentrations tested, there was a reduction in the total activity that brought the activity down to a level that was not significantly different than controls for both the 2.5 and 5 mM PTZ treatment groups (Figures 3A,C). However, larvae pre-treated with either 1.5 or 2 μM THC showed no change in the level of fast activity produced by 2.5 mM PTZ (Figure 3B). When the concentration of THC was increased to 3 μM, there was a significant reduction in the fast activity. Similarly, when the concentration of PTZ was increased to 5 mM, pre-exposure to 1.5 μM of THC again had no effect on the fast activity but increasing the concentration of THC to 2 or 3 μM did reduce the fast activity by approximately 50% (Figure 3D). All three of the concentrations tested also reduced the total activity of control larvae by a similar amount as was found for the PTZ-treated larvae (Figure 3E). This may indicate that, unlike CBD, part of the effect of THC may be due to general sedation. However, when the THC level was increased, the fast activity (but not the overall activity) was further reduced. This would indicate that part of the effect of THC on the PTZ induced activity is specifically on the fast activity. We did not increase the concentration of THC above 3 μM as we have previously found that higher levels of THC lead to an further decrease in the activity of control larvae, which are likely to confound any effect on the PTZ-induced activity patterns (Achenbach et al., 2018).

### Combined Exposure of CBD and THC Appears to Have a Synergistic Effect on the PTZ-Induced Behavior

**Figure 4:**
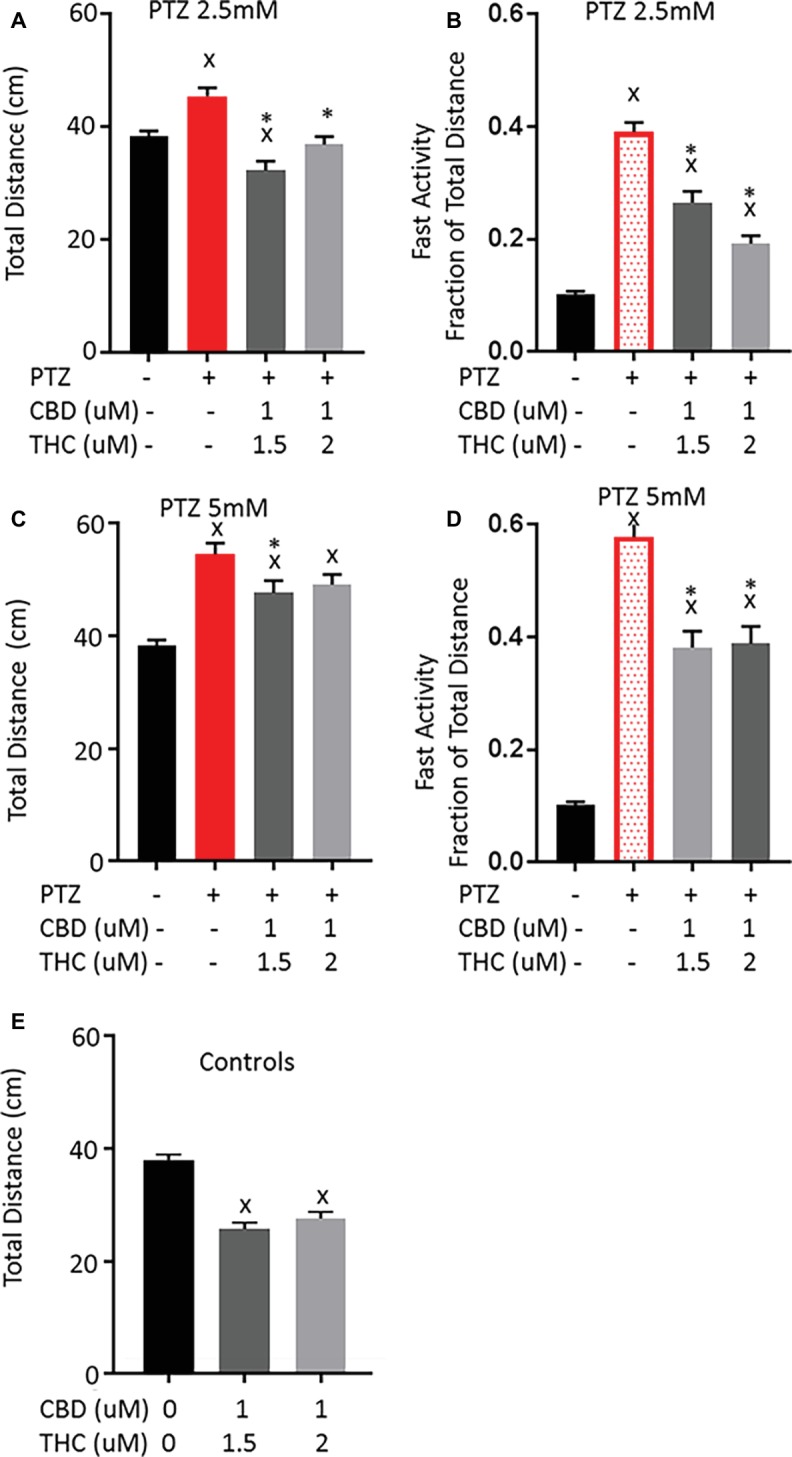
Comparison of PTZ-induced activity patterns following pre-exposure to cannabidiol (CBD) and tetrahydrocannabinol (THC). **(A,C)** Total activity during 30-min time bin following exposure to 2.5 and 5 mM PTZ. **(B,D)** Fast activity (>20 mm/s) represented as a fraction of total activity. **(E)** Control activity following CBD + THC exposure. Significance was measured using a one-way ANOVA followed by Dunnet’s multiple comparison test (*p* = 0.05). X: significant difference from controls.*: significant difference from PTZ (*n* = 36 for each treatment group; *n* = 72 for PTZ 2.5, PTZ 5, and controls).

We have previously demonstrated that co-exposure of larvae to CBD and THC in combination can lead to a synergistic shift in the behavioral response to acute exposure (Achenbach et al., 2018). Thus, we tested the potential effect of combinations of THC and CBD on the PTZ-induced activity patterns. To do so, we chose to combine a number of concentrations, beginning with levels of CBD and THC for which single treatments were not able to prevent PTZ-induced hyperactivity on their own. At 1.5 μM THC, there is no significant effect on the fast activity induced by 2.5 or 5 mM PTZ and 1 μM CBD only produces a small reduction in the fast activity induced by 2.5 mM PTZ without affecting the total activity change. Interestingly, we found that when applied together, 1 μM CBD and 1.5 μM THC pre-exposure led to a significant decrease in both the total and fast activity increase produced by 2.5 and 5 mM PTZ (Figure 4). Similarly, when 1 μM CBD was combined with 2 μM THC, there was a larger reduction in the PTZ-induced activity than either alone at 2.5 mM (Figures 4A,B). However, there was no further reduction in the activity pattern produced by 5 mM PTZ compared to that of THC at 2 μM (Figures 4C,D). Similar to THC alone, when CBD and THC were combined, there was a significant reduction in the activity of control larvae and the reduction in the PTZ-induced total activity brought the activity down to levels similar to controls. This may again suggest that THC produces a general sedation that may partially explain the decrease in the PTZ-induced activity pattern.

### High Doses of THC and CBD Are Necessary to Alleviate *GABRA1*−/− Seizures

**Figure 5:**
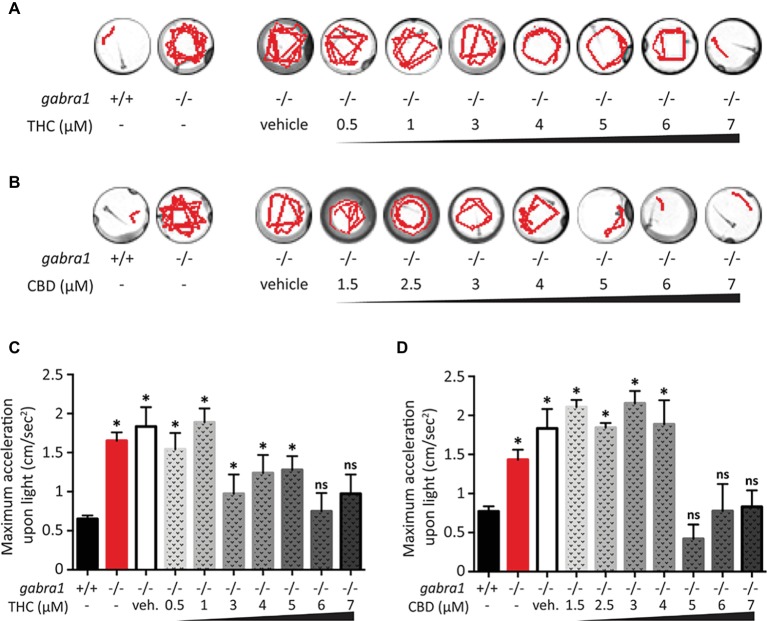
Single exposure of CBD and THC to *GABRA1*−/− epileptic larvae. **(A,B)** Swimming tracks of individual larvae upon light exposure. Unlike *GABRA1+/+* (WT), *GABRA1*−/− (HM) display an increased response to light associated with epileptic seizures. High doses of THC **(A)** or CBD **(B)** prevent this epileptic behavior. **(C,D)** Quantification of the maximum acceleration of the larvae during the 30 s following light exposure. The increase in maximum acceleration is associated with the epileptic seizure. Concentration of THC higher than 6 μM **(C)** and concentrations of CBD higher than 5 μM significantly rescue the *GABRA1*−/− exacerbated acceleration (*t* test vs. WT: * <0.05; *n* > 15).

**Figure 6:**
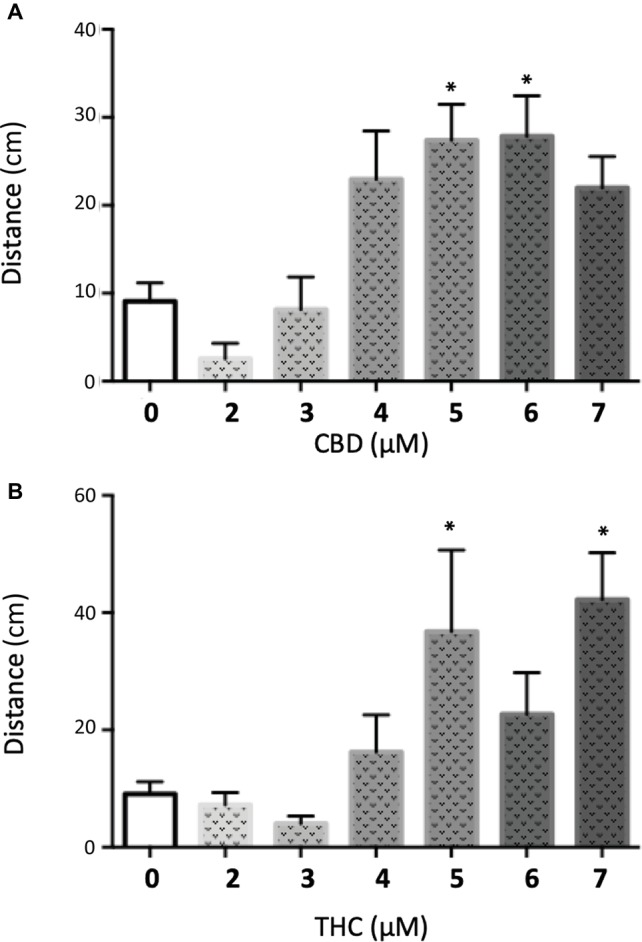
Quantification of the swimming distance of 5 dpf larvae during 90 min after treatment with different concentrations of CBD **(A)** or THC **(B)** (*t* test vs. untreated: * < 0.05; *n* > 15).

We decided to complement our initial findings from the PTZ exposed larvae by testing the effect of CBD and THC on *GABRA1*−/− zebrafish larvae that have been shown to display sporadic neuro-hyperactivity (seizures) beginning at 4 days post-fertilization (dpf). Importantly, the *GABRA1*−/− seizures can also be triggered by a light stimulus, which allows for a controlled generation of seizures (Samarut et al., 2018). The intensity of the seizures can be directly visualized by the swimming tracks of the larvae following light exposure and quantified by the acceleration of the larvae triggered by the light stimulus. We tested the effect of pre-exposure to different concentrations of THC and/or CBD on 5-dpf *GABRA1*−/− larvae by comparing the light-induced acceleration before and after treatment (Figure 5). Based on the results of the PTZ model, we exposed the *GABRA1*−/− larvae to increasing concentrations of both THC and CBD beginning at 0.5 μM until levels were reached that significantly reduced the photo-inducible seizures. We found that THC eliminates the *GABRA1*−/− seizures at a concentration of 6 μM but had no significant effect on the acceleration of the larvae upon light stimulus at concentrations below 6 μM as shown both statistically and through representative swimming tracks (Figures 5A,C). Similarly, CBD was only effective at preventing the seizures at concentrations higher than 5 μM (Figures 3B,D). Since the concentrations required to oppose the *GABRA1*−/− seizures were higher than those previously tested by our group (Achenbach et al., 2018; Ellis et al., 2018) and were higher than those necessary to counter the PTZ-induced activity, we assessed their effect on control larvae. We found a bimodal concentration-dependent activity pattern where low doses of either CBD or THC decreased the general activity of the fish, while concentrations higher than 3 μM led to hyperactivity (Figure 6). This is consistent with our previous work evaluating acute exposure to CBD and THC where we found that as the concentration of either compound was elevated the behavioral pattern produced included intermittent periods of hyperactivity along with periods of inactivity that increased during prolonged periods of exposure (Achenbach et al., 2018). It thus appears that the higher concentrations of THC or CBD tested in this study induce changes in the basal activity that should be considered when evaluating their potential anti-seizure effects.

### Combined Exposure to THC and CBD Produce Synergistic Activity Against Genetic Seizures

**Figure 7:**
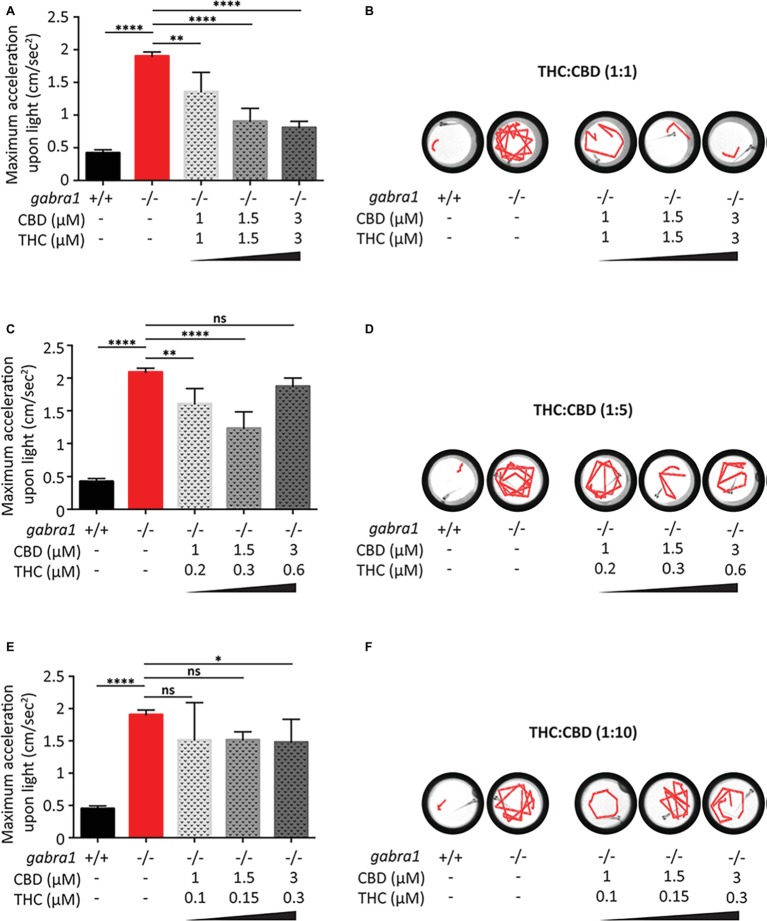
Combined exposure of CBD and THC to *GABRA1*−/− epileptic larvae. Quantification of maximum acceleration **(A,C,E)** and swimming tracks upon light **(B,D,F)** following exposure to different concentrations of THC/CBD at a ratio of 1:1 **(A,B)**, 1:5 **(C,D)**, or 1:10 **(E,F)** (one-way ANOVA: **** <0.0005, ** < 0.005, * < 0.05; *n* > 15).

Similar to the PTZ model, we tested the effects of co-exposure to CBD and THC on the minimum concentration required to oppose the *GABRA1*−/− seizures (Figure 7). Based on the results of the PTZ testing, we exposed the *GABRA1*−/− larvae to different concentrations of CBD and THC beginning at a 1:1 ratio of THC to CBD (Figures 7A,B). Interestingly, we found that at 1.5 and 3 μM THC/CBD (1:1), there was a significant opposition to the *GABRA1*−/− seizures, as quantified by the acceleration upon light and by visualization of the swimming tracks upon light. We then reduced the concentration to 1 μM THC/CBD (1:1) and found that although it significantly reduced the maximum acceleration value compared to untreated *GABRA1*−/− larvae, this combination could not prevent the seizure-like behavior (Figures 7A,B). Finally, since the psychoactive effects of THC are one of the major deterrents to the use of cannabis as a therapeutic, we evaluated whether the concentration of THC could be lowered and still provide a relevant level of synergy with CBD to prevent the *GABRA1*−/− seizures. We then exposed mutant larvae to 1:5 and 1:10 ratios of THC:CBD at the same concentrations of CBD tested at the 1:1 ratio (Figures 7C–E). While at 1.5 μM CBD the 1:5 ratio showed a partial effect on the *GABRA1*−/− seizures, we found that neither of the ratios tested provided the same level of opposition to the *GABRA1*−/− seizures as the 1:1 ratio.

It thus appears that the synergistic effect between THC and CBD is only evident at a 1:1 ratio and a minimum concentration of THC:CBD of 1.5 μM is required to prevent the seizures of the *GABRA1*−/− larvae. Importantly, the combination of THC and CBD at the 1:1 ratio resulted in more than a threefold reduction in the concentration required to oppose the seizures, which brings the concentrations of THC and CBD down to a level that has less of an effect on control activity.

## Discussion

The development of new therapeutics for the treatment of neuronal hyperactivity has largely stalled with no major advances over the last 40 years. In fact, in the context of epilepsy, more than a third of newly diagnosed cases still fall into the anti-epileptic drug (AED) resistant category (Mohanraj and Brodie, 2005). The development of new compounds that can potentially treat individuals with multi-drug resistant epilepsy provides a substantial challenge that requires both new sources of prospective therapeutics and more informative models with which to test said compounds. In the current study, we have made use of two distinct neuro-hyperactivity models as previously developed with zebrafish larvae to test the efficacy of the phytocannabinoids THC and CBD for the treatment of seizures. We found that both cannabinoids opposed the behavioral hyperactivity induced by the PTZ exposure and the *GABRA1*−/− genetic model. However, the concentration required to alleviate the genetic seizure activity was somewhat higher (at least threefold). We also found that when THC and CBD were applied together, they displayed a synergistic effect. This was particularly important for the *GABRA1*−/− larvae as it brought the concentrations of THC and CBD required to oppose the seizure activity to levels that had much less of an effect on baseline larval behavior.

The use of cannabinoids for the treatment of seizures is not novel and is in fact one of the oldest reported uses of cannabis (Friedman and Sirven, 2017). However, the pharmacology of both THC and CBD is complex with both compounds exerting their effect through multiple pathways. This is particularly true for THC, which not only exerts its primary anti-seizure effect through the CB1 receptor but also has a number of other potential targets that may be linked to its anti-seizure properties (Gaston and Friedman, 2017). While this may aid in the efficacy of THC for the treatment of seizures, the consequences of activating these numerous potential targets are not well understood. This may be linked to the inconsistent findings from different studies that have evaluated the efficacy of THC for the treatment of epilepsy. As was shown in this study, the pharmacology of THC is complex producing multi-modal concentration-dependent effects, which further complicates evaluating its efficacy as a therapeutic. CBD also has numerous targets that may be linked to its anti-seizure properties, but the exact mechanisms are less clear (Wallace et al., 2001; Wallace et al., 2003; Gaston and Friedman, 2017; Gaston and Szaflarski, 2018). An additional complexity of using CBD for the treatment of epilepsy stems from its inhibition of CYP enzyme activity, which can have profound effects on the metabolism of other AEDs (Jiang et al., 2013). An example of this is the interaction of CBD with clobazam where co-administration was shown to lead to an increase in the levels of clobazam that was accompanied by a subsequent increase in sedation (Geffrey et al., 2015; O’Connell et al., 2017). This is important since patients with multi-drug resistant epilepsy generally undergo polypharmacy and CBD or cannabis are rarely used alone in the clinical setting but rather are used as an adjunct to anti-epileptics that are currently used by the patient (Loscher and Schmidt, 2011; Brodie et al., 2015; Brodie and Ben-Menachem, 2018). This issue is further confounded by the fact that many patients are using cannabis as an adjunct to their current therapeutic as opposed to one of the purified cannabinoids. Cannabis is comprised of over 70 different cannabinoids that may contribute to its various effects through numerous mechanisms of action and drug interactions (Boggs et al., 2016). In the current study, we have demonstrated the ability of zebrafish larval seizure models to act as a platform to test the effects of different drug combinations on the seizure models while at the same time evaluating potential negative effects, such as an increased level of sedation.

Pentylenetetrazole (PTZ) is a γ-aminobutyric acid (GABA) antagonist and one of the most widely studied pro-convulsing chemicals (Kundap et al., 2017). Along with the maximal electroshock seizure test (MES), it is considered one of the gold standard rodent models for the identification of drugs with anti-seizure potential and previous studies have used it to study the effects of anticonvulsants on zebrafish (Baraban et al., 2005; Baraban, 2007; Berghmans et al., 2007; Loscher and Schmidt, 2011; Ellis and Soanes, 2012). Similar to rodent assays, PTZ produces seizures in both adult and larval zebrafish. Exposure of larvae to PTZ has been shown to produce a multi-stage concentration-dependent behavioral response characterized by an increase in fast-darting activity and clonic activity that is not normally found for control larvae (Baraban et al., 2005; Ellis et al., 2012; Ellis and Soanes, 2012). While the fast-darting activity precedes the clonic state and subsequent loss of posture, it is still considered a part of the seizure-like behavior and as such provides a quantifiable measure that can be used to assess the potential efficacy of novel therapeutics (Griffin et al., 2018). The other model used in the current study is the recently developed *GABRA1*−/− genetic knockout model (Samarut et al., 2018). This model produces sporadic seizures in juvenile fish characterized by intense convulsions, loss of swimming posture, and repetitive jerks of the jaws that are indicative of tonic-clonic-like seizures. These events are accompanied by a more global elevation in neural activity than what is found for the PTZ-induced models, supporting the more severe nature of the events. Since the concentration of PTZ used in this study was not high enough to lead to clonic activity, it could be considered a non-convulsive model of neuro-hyperactivity, while the *GABRA1−/−* model may represent generalized tonic-clonic seizures. The more severe nature of the *GABRA1−/−* model is highlighted by the fact that the concentration of THC or CBD that was required to oppose the *GABRA1*−/− seizures was three- to fourfold higher than the level that had an effect on the PTZ-induced darting events. Interestingly, the requirement for a higher level of THC and CBD to oppose the *GABRA1*−/− seizures was overcome by combining THC and CBD. At a 1:1 ratio, the level of CBD and THC required to reduce the seizure activity dropped from 5–6 μM to between 1.5 and 3 μM. This is an important finding since exposure of larvae to either compound at levels above 5 μM produces several negative behavioral effects that include hyperactivity and more sporadic movements that are likely indicative of an increased side-effect profile at these levels. At ratios of THC:CBD below 1:1, there did not appear to be the same synergistic effect on the *GABRA1−/−* model, which may indicate that there is a minimum level of THC required to achieve the anticonvulsant activity. Additionally, there did not appear to be as large as that of a synergistic effect of THC and CBD on the PTZ-induced hyperactivity. This may be the result of the lower overall level of neural hyperactivity associated with the PTZ model. In fact, the neural hyperactivity induced by PTZ exposure at the concentrations tested has previously been shown to be regionalized within the brain of the larvae (Ellis et al., 2012). Opposing this localized neural hyperactivity appears to require a lower level of either cannabinoid on their own and may not benefit from the synergistic effect to the same degree as the *GABRA1−/−* model. Future work is required to analyze the pathway specificity of the cannabinoid activity and to assess the mechanism of action of the synergistic effect.

While it has been shown that in a clinical setting CBD (Epidolex) can be effective against Dravet syndrome anecdotally, there are suggestions that products with some THC are better at controlling seizures than CBD alone (Mechoulam, 2017). However, controlling the levels of THC that are required to treat seizures is important as it has been demonstrated that THC can produce cognitive and psychiatric side effects (Volkow et al., 2014). In this context, we sought to evaluate the minimal level of THC required to show a synergistic effect. Lowering the ratio of THC:CBD below 1:1 resulted in a decrease in the effectiveness of the mixture against the *GABRA1*−/− seizures. The efficacy of the 1:1 ratio is in line with the pharmaceutical preparation Sativex (1:1 ratio THC:CBD), which is the first cannabis preparation to win market approval. This ratio has been shown to be effective in treating several symptoms of multiple sclerosis, neuropathic, and cancer pain along with rheumatoid arthritis (Boggs et al., 2016). The presence of CBD may help to prevent some of the side effects associated with CBD as it has been suggested that the CBD antagonism of the CB1 receptors in the CNS reduces the effect of THC on the CB1 receptor, thus damping its psychoactivity, while still allowing it to exert an effect on other targets such as a peripheral effect through the CB2 receptor (Russo and Guy, 2006; Boggs et al., 2016). Additionally, it has been shown that CBD can inhibit the hydroxylation of THC to its primary psychoactive metabolite 11-hydroxy-THC, which may also help to reduce its psychoactive effects (Bornheim et al., 1995; Nadulski et al., 2005; Klein et al., 2011; Britch et al., 2017; Hlozek et al., 2017). Targeting the endocannabinoid system with potential therapeutics is not without its perils as was found for the CB1 antagonist Rimonabant, which was discontinued due to its psychiatric side-effect profile. Since zebrafish also provide a well-established model for toxicity testing, they provide a platform with which to test both the potential efficacy and the potential toxicity of novel anti-seizure compounds such as the cannabinoids tested in this study.

The testing of CBD and THC against the two models of seizure-like activity has shown their ability to oppose behavioral changes linked to neuro-hyperactivity. While the behavioral patterns produced by both models have been shown to be linked to increases in neural activity indicative of seizures, care must be taken when evaluating the anti-seizure effects of drugs using only behavioral readouts. In the current study, there were indications that the changes in behavior induced by CBD and THC do indicate an opposition to the increased neuro-hyperactivity found for each model. This is highlighted by the fact that CBD did not appear to show a large sedative effect at the concentrations required to oppose the behavioral hyperactivity. Conversely, THC did show a decrease in control behavior that may indicate some level of general sedation as opposed to a direct anti-seizure effect. Further work is required in order to confirm the anti-seizure mechanism of action of both compounds, which may help to define their potential efficacy. Importantly, the testing conducted in the current study provides an initial platform to not only test the potential anti-seizure effects of other cannabinoids and cannabis extracts, but it can also be used to test their interaction with known AEDs. Information on these types of interactions will be important when moving from lab testing to clinical trials.

## Data Availability

The datasets generated for this study are available on request to the corresponding author.

## Author Contributions

ÉS, JN, and LE designed the project. ÉS, JN, UK, and LE performed the experiments. ÉS, JN, UK, PD, and LE analyzed the data. ÉS, JN, and LE drafted the manuscript.

### Conflict of Interest Statement

ÉS is a co-founder of Modelis inc.

The remaining authors declare that the research was conducted in the absence of any commercial or financial relationships that could be construed as a potential conflict of interest.
